# Assessment of HIV-related stigma in a US faith-based HIV education and testing intervention

**DOI:** 10.7448/IAS.16.3.18644

**Published:** 2013-11-13

**Authors:** Jannette Y Berkley-Patton, Erin Moore, Marcie Berman, Stephen D Simon, Carole Bowe Thompson, Thomas Schleicher, Starlyn M Hawes

**Affiliations:** 1 Department of Psychology, University of Missouri – Kansas City, Kansas City, MO, USA; 2 Department of Psychology, Stetson University, DeLand, FL, USA; 3 Department of Biomedical and Health Informatics, University of Missouri – Kansas City, Kansas City, MO, USA; 4 Department of Psychiatry and Behavioral Sciences, University of Washington School of Medicine, Seattle, WA, USA

**Keywords:** faith-based settings, faith organizations, HIV-related stigma, African American church, church members, community members, HIV intervention, HIV testing, people living with HIV

## Abstract

**Introduction:**

The African American church is a highly influential institution with the potential to greatly increase the reach of HIV prevention interventions and address HIV-related stigma in US African American communities. However, there are few studies on HIV-related stigma and African American church populations. This study explored HIV-related stigma among church and community members participating in an HIV education and testing intervention pilot study in African American churches, named Taking It to the Pews.

**Methods:**

Four African American churches located in Kansas City, MO and KS, were randomized to either intervention or comparison groups. Churches assigned to the intervention group received religiously tailored HIV education, testing and compassion messages/activities (e.g., sermons, brochures/church bulletins, testimonials) via the Taking It to the Pews HIV Tool Kit. Comparison churches received non-religiously tailored HIV information. HIV-related stigma was assessed with 543 church members and with community members served through church outreach services (e.g., food/clothing pantries, social services) in the four churches. Participants completed surveys at baseline, 6 months and 12 months to assess their HIV-related stigma beliefs, exposure to intervention components and satisfaction with the study.

**Results:**

At baseline, HIV-related stigma beliefs were similar across experimental groups and were quite low. Mean HIV-related stigma scores were not significantly different between experimental groups at 6 months (*p*=0.92) or at 12 months (*p*=0.70). However, mean HIV-related stigma scores within both groups showed decreasing trends at six months, which approached significance. Analysis of previously studied HIV-related stigma factors (e.g., age, gender, income, HIV knowledge, religiosity) did not yield changes in the null findings. Intervention group participants were highly exposed to several intervention components (sermons, HIV resource tables, posters, brochures/church bulletins). Overall, participants were highly satisfied with the intervention pilot study.

**Conclusions:**

African American churches may be well positioned to increase the reach of HIV prevention interventions to church and community members and could serve an important role in addressing HIV-related stigma in their church communities. Future research is needed on measuring HIV-related stigma beliefs and on testing intensive, scalable, religiously tailored HIV interventions to impact HIV-related stigma in African American churches.

## Introduction

HIV continues to disproportionately impact African American (AA) communities in the United States [1–5]. A primary barrier that impedes efforts to develop, implement and test HIV education, testing and linkage-to-care programmes is HIV-related stigma (hereafter referred to as “HIV stigma”) [6–11], which can hamper efforts to reduce the HIV burden in AA communities. Studies have shown that fear of HIV stigma has been associated with reduced rates of HIV testing and engagement in treatment among AAs [12, 13], and poor disease management and quality of life for AA people living with HIV (PLHIV) [12–15].

Traditionally, stigma has been defined as negative attitudes towards preventable or controllable illnesses with causes identified as undesirable/immoral behaviours (e.g., having sex outside of marriage) and associated with certain groups (e.g., men who have sex with men) who are blamed for their illness [16]. Goffman's early work on stigma suggested that negative attitudes towards undesirable behaviours arise from perceptions that “out-groups” exhibiting these unacceptable behaviours have violated a community's set of values or community norms [17]. Regarding HIV stigma, PLHIV may be prone to experience HIV stigma based on others’ perceptions of these behaviours, which can lead to perceived and actual disadvantage or discrimination. Parker and Aggelton [18] argue for the need to go beyond the individual focus on HIV stigma and to instead examine stigma as an evolving social process with consideration given to changing the structural and changing beliefs of stigma-generating groups through community mobilization efforts. However, important questions remain on how community mobilization in settings that traditionally may have promoted HIV stigmatizing beliefs can now be engaged in positively influencing their constituents to extend compassion and support for PLHIV, join PLHIV in speaking against HIV stigma, and ultimately encourage their community members to assist in advocacy efforts to eliminate injustices and discrimination against PLHIV.

The AA church is a long-standing, powerful institution with a tradition of mobilizing AA communities for social and political change and could play an important role in leading mobilization efforts to reduce HIV stigma beliefs among AAs. Nationwide studies indicate that most AAs in the United States attend church weekly [19–21] and believe that church leaders are highly influential [22, 23]. Also, most AA churches have similar characteristics, including similar modes of worship (e.g., sermons, testimonials) [23, 24], and community outreach ministries [23–25] that could facilitate the implementation of HIV prevention interventions to educate about HIV risks, promote HIV testing and impact HIV stigma in their church communities. Although the AA church has been criticized for its lack of involvement in the early years of the HIV epidemic [7], a shift in churches willing to address HIV and participate in HIV-related research studies seems to be emerging [24, 26–30]. Still, several of the controversial issues (e.g., homosexuality, premarital sex, multiple sex partners, drug use) associated with HIV stigma in AA communities are not discussed or are denounced in many AA churches [31–33]. Studies with AA faith leaders suggest they are interested in participating in church-based HIV prevention interventions [24, 31, 32], but also identify HIV stigma as a key barrier that can slow adoption of such interventions [33–35]. Recent studies have found that with religiously tailored HIV education and motivational supportive strategies, the AA church can serve as a potentially influential venue to address HIV and related stigma beliefs among their church/community members [24, 26–30].

Few studies have examined HIV stigma beliefs with AA church populations [35, 36]. Still, these studies with AA church populations found low levels of HIV stigma beliefs and related personal factors, such as age, education, religiosity and HIV knowledge [35, 36]. Yet, to our knowledge, no studies have examined HIV prevention intervention effects on HIV stigma in AA churches. The primary aim of this study was to pilot test feasibility of the Taking It to the Pews (TIPS) project (an HIV education and testing intervention in AA churches) and determine its effect size on HIV-testing rates to plan a future, clustered randomized trial. During this pilot study, we also assessed HIV stigma beliefs as an intervention outcome. The current study reports on HIV stigma beliefs assessed at baseline, 6 months and 12 months in the pilot study.

## Methods

### Contextual background

The TIPS project used a community-based participatory research (CBPR) approach to mobilize AA churches to address HIV prevention through education and testing [37]. AA church leaders chose this TIPS research focus with the aim of increasing HIV testing (instead of focusing on condom use as an HIV prevention strategy) and reducing HIV stigma. Church leaders along with church and community members (inclusive of PLHIV) participated in TIPS intervention development (including creation of the TIPS HIV Tool Kit), implementation and evaluation [24, 27, 38]. Using the CBPR approach, trained church leaders delivered religiously appropriate TIPS HIV Tool Kit materials/activities through multiple church outlets (community outreach ministries, church services, group ministries, peer-to-peer). Tool kit materials/activities (e.g., sermon guides, posters, resource tables, video/printed/in-person testimonials, church bulletin inserts, brochures) were designed to: (a) “fit” within existing church activities for ease in mobilizing AA churches, (b) provide HIV education and enhance compassion/respect for PLHIV and (c) engage pastors to promote HIV testing and stigma reduction [24]. Based on past AA church population studies [35, 36], HIV stigma was hypothesized to be related to age and religiosity; and inversely related to income, HIV knowledge and intervention exposure.

### Study design

Pastors of five AA churches were approached for recruitment; four agreed to have their churches participate in the study, and one declined due to commitments to other new projects. Churches were matched on size of membership and type of outreach services and were randomly assigned to intervention (*n*=2 churches) and comparison groups (*n*=2 churches). Intervention churches received one to two TIPS intervention religiously tailored materials/activities, and comparison churches received one to two non-religiously tailored HIV informational brochures/church bulletins, monthly. Participants were assessed at baseline, 6 months and 12 months.

### Setting and participants

The four participating churches were located in Kansas City, MO and KS, USA. Churches were recruited to participate based on four criteria: (a) minimum of 150 AA adult church members; (b) a willing pastor and two church members serving as church liaisons to assist in delivery of TIPS study activities; (c) outreach services (e.g., food/clothing programmes, recovery programmes) available to a minimum of 50 community members per month; and (d) never having hosted an HIV-related event. Participating churches were compensated $2500 for recruitment, retention, implementation of TIPS interventions and reporting data through an online system. Church liaisons were provided with $500 for their assistance in intervention delivery. Additionally, churches were supplied with technology support to assist in delivering the intervention (e.g., digital projector, telephone messaging system). Participating church members and community members (who regularly used church outreach services) were aged 18–64 and were consented to participate in the study, and received $10 for completing baseline, 6-month and 12-month surveys ($30 total). Surveys were administered to church members after their church services and to community members after church community outreach activities. The University of Missouri – Kansas City IRB approved the study.

### Intervention overview

Over a 12-month period, church liaisons delivered the TIPS HIV Tool Kit materials/activities through various church activities (e.g., community outreach, church services, ministry groups, interpersonal interactions). The original TIPS case study and all HIV Tool Kit materials/activities have been fully described elsewhere [24, 27] and are briefly described here.

#### Community outreach level activities

Church liaisons delivered printed materials (e.g., brochures, printed testimonials) to community members through church outreach services (e.g., food/clothing pantry, social services). At opportune times, pastors delivered brief messages about HIV topics and promoted HIV testing and PLHIV compassion with community members where/when appropriate (e.g., parents meetings, before prayer at a free meal event). In collaboration with local health agencies, liaisons coordinated three HIV-testing events for church/community members. At least one of these testing events was scheduled during a highly attended community outreach activity (e.g., food/clothing programme, social services).

#### Church-wide service level

Pastors delivered sermons on HIV topics, promoted HIV testing and encouraged the reduction of HIV risks and stigma. During HIV-testing events, pastors modelled receipt of HIV testing for church/community members. Also, HIV information was delivered through church bulletins/brochures, announcements, responsive readings, and through in-person and printed testimonials from PLHIV and from members who had been tested for HIV.

#### Ministry group level

Printed/video testimonials of members who had been tested for HIV with accompanying discussion guides, HIV education games (HIV Basics Jeopardy, Wheel of Awareness, HIV Testing Jeopardy) with facilitator instruction guides and printed Tool Kit materials were delivered through women's, men's and singles ministry group meetings.

#### Interpersonal/individual-level activities

Church/community members received brochures tailored by gender with information on HIV risks, prevention and testing. They also received scripted phone voice/text messages read by pastors and church liaisons to remind them about upcoming HIV-testing events and to increase intentions to seek HIV testing.

### Measures

#### Participant characteristics

Demographics (e.g., age, gender, income) and HIV testing (ever; yes/no) were assessed. Religiosity was measured with a summation of the seven-item version of the Religious Background and Behavior Scale [39] with six items on participants’ past year engagement in religious activities (e.g., prayed, attended a church service) using an eight-point Likert scale (0=never to 7=always) and one item on description of their religiosity (atheist=0 to religious=4) (*α*=0.77). HIV knowledge consisted of the summation of correct scores for 10 items (e.g., “You can get HIV if you share a drink, sink, shower, or toilet seat with someone who has AIDS”; *α*=0.56) from the HIV Knowledge Questionnaire [40].

#### HIV stigma beliefs

Similar to other stigma studies with church populations [35, 36], HIV stigma items were selected from national studies on HIV stigma [6, 41] and based on pre-intervention focus group findings [42]. The following five items assessed HIV stigma: (a) “How *comfortable* would you be sharing a pew with an HIV-positive person?” (symbolic contact); (b) “How strongly would you agree or disagree that scientists and doctors can be *trusted* to tell the truth about HIV?” (trust of authorities); (c) “How *afraid* are you of people who are infected with HIV?” (fear); (d) “If you were going to be tested for HIV, how *concerned* would you be that you might be treated differently or discriminated against if your test results were positive for HIV?” (discrimination); and (e) “How strongly would you agree or disagree that HIV-positive people are *responsible* for their illness?” (attitudes towards PLHIV) using a four-point Likert scale (e.g., 1=not at all afraid to 4=very afraid). A mean HIV stigma score was computed from items 1–4. The fifth stigma item (*responsible*) was not included in the final analyses of mean HIV stigma scores after preliminary analyses indicating poor reliability (*α*=0.37). After removing the *responsible* item from the mean HIV stigma scores, the *α*'s ranged from 0.50 to 0.55 across assessments.

#### Intervention exposure and satisfaction

Intervention exposure was assessed on participants’ exposure to 11 TIPS materials/activities (e.g., pastoral sermons, brochures/church bulletins, PLHIV/others’ testimonials, resource tables, health educator presentations) (1=yes; 0=no). Intervention participants’ satisfaction was assessed (e.g., how clearly HIV information on HIV delivered, how compassionately their pastor spoke about HIV) on a seven-point scale (1=not at all satisfied to 7=very satisfied).

### Data analysis

Frequencies and means were computed to describe participant characteristics, individual HIV stigma items, and intervention exposure and satisfaction. Analyses of mean HIV stigma scores (study outcome variable) were based on randomized churches, instead of individuals. Mean HIV stigma scores were examined using a mixed linear regression model (IBM SPSS version 20 and R version 2.12.1), which accounted for subject non-independence within church. The model included experimental condition and covariates (age, gender, income, religiosity, knowledge) as fixed-effect terms; churches nested in experimental condition were included as random effects terms. The mixed linear regression model found a 0 intracluster correlation within churches at baseline, 6 and 12 months; therefore, a simple linear regression model without clustered churches was used. Linear regression analysis was also conducted to determine if increased intervention exposure (dosage) was related to an increase in mean HIV stigma scores in the intervention group. Due to the significant attrition of participants across groups at 6 and 12 months, results were examined using simple mean imputation. Imputation yielded results similar to using complete case analysis; therefore, results on mean HIV stigma scores are reported from complete case analysis with individuals who completed questionnaires at baseline and in each subsequent time point.

## Results

### Participant characteristics

**Figure 1:**
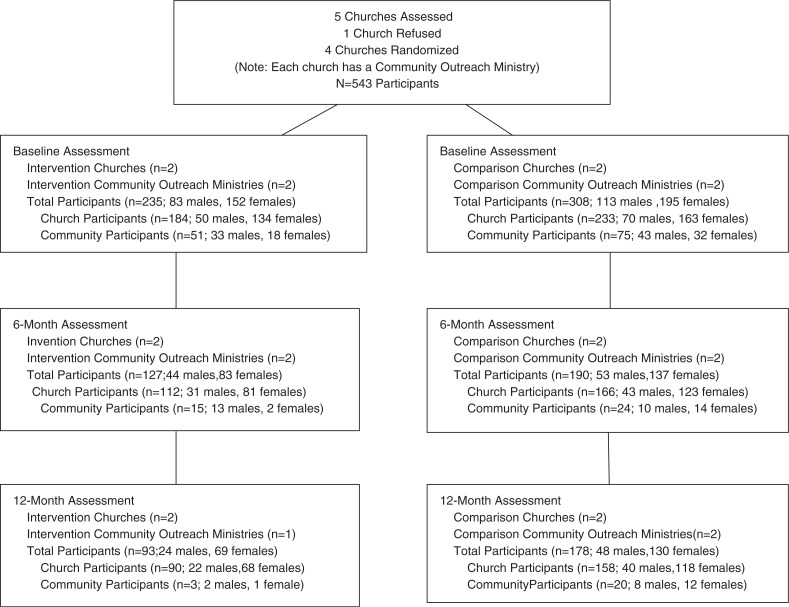
Flow of churches and participants through completion of 12-month assessment: Taking It to the Pews Pilot Study: Kansas City Metropolitan Area, USA.

Overall, 543 church/community members were recruited from the four churches at baseline (*n*=235 intervention participants; *n*=308 comparison participants), as shown in Figure 1. Six-month and 12-month retention rates were 54% (*n*=127) and 40% (*n*=93), respectively, for the intervention group, and 62% (*n*=190) and 58% (*n*=178), respectively, for the comparison group. Participants were primarily Baptist (36%, *n*=196) and Church of God in Christ (32%, *n*=175) and had a mean age of 42.3 (SD=13.5). Also, most participants were female, single, highly religious (85% prayed daily; 79% attended church weekly) and had been tested for HIV, as shown in Table 1. The overall average HIV knowledge score at baseline was 7.49. Most frequently incorrect HIV knowledge items were: “A condom should be completely unrolled before it is placed on the penis” and “A person can get HIV by giving blood.” Except for sexual identity, intervention and comparison arm characteristics were similar. For HIV stigma and exposure measures, there were no differences between 6- and 12-month completers and non-completers on baseline data. Differential attrition occurred at 6 and 12 months on demographic measures; non-completers tended to be younger, male, have less education and income, and be community members.

**Table 1:** Baseline participant characteristics

	Intervention group			Comparison group			
			
Survey measures	Church members	Community members	Overall	Church members	Community members	Overall	*p*
Age							0.27
18–29	25.1% (46)	23.1% (12)	24.7% (58)	22.6% (54)	17.3% (13)	21.3% (67)	
30–49	39.3% (72)	36.5% (19)	38.7% (91)	37.7% (90)	41.3% (31)	38.5% (121)	
50–64	34.4% (63)	40.4% (21)	35.7% (84)	36.8% (88)	41.3% (31)	37.9% (119)	
Gender							0.78
Male	27.3% (50)	63.5% (33)	35.3% (83)	29.3% (70)	57.3% (43)	36.0% (113)	
Female	72.7% (133)	36.5% (19)	64.7% (152)	68.2% (163)	42.7% (32)	62.1% (195)	
Sexual identity							0.01
Heterosexual	84.7% (155)	65.4% (34)	80.4% (189)	89.5% (214)	80% (60)	87.3% (274)	
Homosexual	1.1% (2)	–	0.9% (2)	2.1% (5)	4% (3)	2.5% (8)	
Bisexual	–	7.7% (4)	1.7% (4)	0.4% (1)	2.7% (2)	1.0% (3)	
Other/choose not to Answer	11.4% (21)	25.0% (13)	14.5% (34)	3.7% (9)	12% (9)	5.7% (18)	
Marital status							0.73
Single/separated/divorced/widowed	56.8% (104)	82.7% (43)	62.6% (147)	52.7% (126)	81.3% (61)	59.6% (187)	
Co-habiting/married	42.7% (78)	17.3% (9)	37.0% (87)	44.8% (107)	17.4% (13)	38.3% (120)	
Monthly income							0.10
$0–$1000	9.8% (18)	50.0% (26)	18.7% (44)	8.8% (21)	46.7% (35)	17.8% (56)	
$1001–$2000	14.2% (26)	5.8% (30)	12.3% (29)	18.4% (44)	18.7% (14)	18.5% (58)	
$2001–$2500	7.7% (14)	1.9% (1)	6.4% (15)	13.0% (31)	5.3% (4)	11.1% (35)	
$2501–$3000	14.8% (27)	7.7% (4)	13.2% (31)	11.7% (28)	4.0% (3)	9.9% (31)	
More than $3000	42.6% (78)	13.5% (7)	36.2% (85)	37.7% (90)	8.0% (6)	30.6% (96)	
Don't know	9.8% (18)	21.2% (11)	12.3% (29)	7.9% (19)	14.7% (11)	9.6% (30)	
Ever tested for HIV							0.12
Yes	71.2% (131)	74.5% (38)	71.9% (169)	72.8% (174)	86.7% (65)	76.1% (239)	
No	27.7% (51)	23.5% (12)	26.8% (63)	24.7% (59)	9.3% (7)	21% (66)	
Religiosity (M, SD) (possible range 0 to 46)	36.9 (7.3)	29.0 (10.8)	35.4 (8.6)	34.5 (7.4)	32.2 (10)	33.9 (8.2)	0.23
HIV knowledge (M, SD) (possible range 1 to 10)	7.4 (1.8)	7.2 (1.6)	7.4 (1.8)	7.7 (1.6)	7.2 (1.8)	7.6 (1.7)	0.36

Note: Percentages are based on actual rather than valid percent. Many of the variables reported in this table had missing data (ranging from 0 to 55), including those who did not respond because the question(s) were not applicable to them (ranging from 0 to 78).

### HIV stigma belief items and scores

Three out of the five HIV stigma items (sharing pew, trust doctors, afraid of PLHIV) were low at baseline (range: 1.59–2.04), as shown in Table 2. Although most of the stigma items showed reductions over time, none were significantly lower at 6 and 12 months. As shown in Table 3, HIV stigma mean did not differ at baseline between experimental groups (*p=*0.24). At 6 months, the difference in mean HIV stigma scores between experimental groups was not significant (*p*=0.92). However, the difference in mean HIV stigma scores within both groups at six months approached significance. At 12 months, the difference in mean HIV stigma scores between groups was not significant (*p*=0.70). Inclusion of age, gender, income, religiosity and HIV knowledge did not change non-significant differences between intervention and comparison group HIV stigma change scores at 6 and 12 months. However, linear regression with the baseline mean HIV stigma score as the outcome identified two predictors: HIV knowledge (*β*=−0.09, *p*=0.00) and income level (*β*=−0.19, *p*=0.04), meaning that increased HIV knowledge and higher levels of income were predictive of lower HIV stigma at baseline.

**Table 2:** HIV stigma items

	Intervention, M (SE)	Comparison, M (SE)	*P*
Comfortable sharing pew			
Baseline	1.59 (1.01)	1.78 (1.08)	0.10
6 months	1.45 (0.89)	1.63 (1.03)	0.91
12 months	1.68 (1.13)	1.69 (1.01)	0.37
Trust doctors are telling truth			
Baseline	2.01 (0.88)	2.04 (0.89)	0.78
6 months	1.95 (0.79)	2.01 (0.80)	0.82
12 month	1.97 (0.83)	2.00 (0.85)	0.92
Afraid of PLHIV			
Baseline	1.60 (0.84)	1.66 (0.90)	0.48
6 months	1.51 (0.80)	1.60 (0.77)	0.93
12 months	1.43 (0.77)	1.58 (0.80)	0.95
Concern discrimination			
Baseline	2.45 (1.03)	2.48 (0.99)	0.85
6 months	2.33 (0.96)	2.37 (1.01)	0.75
Post-test	2.31 (0.96)	2.41 (1.03)	0.87
People living with HIV responsible for illness			
Baseline	2.42 (0.87)	2.42 (0.85)	0.62
6 months	2.30 (0.88)	2.42 (0.83)	0.65
12 months	2.23 (0.88)	2.37 (0.80)	0.94

**Table 3:** Mean HIV stigma scores

	Intervention group			Comparison group			Between group change	
	
Outcome	Baseline (*n*=228), mean (SE)	6 Months (*n*=124), mean (SE)	12 Months (*n*=92), mean (SE)	Baseline (*n*=297), mean (SE)	6 months (*n*=181), mean (SE)	12 Months (*n*=172), mean (SE)	6 Months, *p*	12 Months, *p*
HIV stigma scores	1.92 (0.61)^a^	1.84 (0.55)^b^	1.91 (0.62)^c^	1.98 (0.59)	1.90 (0.58)^d^	1.93 (0.62)^e^	0.92	0.70

a Differences between groups at baseline: *p =*0.24.

b Differences within groups: *p=*0.09

c *p=*0.83

d *p=*0.08

d *p=*0.32.

### Intervention exposure

At 12 months, intervention group participants reported exposure to 72% (8 out of 11) of TIPS tools. Highly reported exposure to TIPS materials/activities included sermons (93%), posters (91%), resource tables (90%) and brochures/church bulletins (85%). Several intervention materials/activities were significantly related to (or trending towards) lower HIV stigma items at 12 months. Exposure to health professionals and PLHIV sharing HIV information and HIV-testing events were related to increased comfort in sharing pews with PLHIV (*p*=0.06 and *p*=0.07, respectively). Brochures/church bulletins and pastor-delivered sermons were related to decreased fear of PLHIV (*p=*0.078 and 0.01, respectively). Increased exposure to the intervention was not significantly related to reductions in mean HIV stigma scores at 6 and 12 months (*p=*0.21 and *p=*0.20, respectively).

### Participant satisfaction

Overall, intervention participants reported being highly satisfied with how clear HIV information was delivered (90%), how compassionately their pastor discussed HIV (81%) and how often HIV information and events were offered (80%).

## Discussion

This study examined HIV stigma as an outcome in an HIV education and testing pilot intervention implemented in AA churches. To our knowledge, this the first HIV prevention intervention study to assess HIV stigma outcomes among an AA church population, inclusive of church and community members. Overall, participants were highly religious (e.g., 79% attended church weekly), thus highlighting the potential reach and influence of churches to deliver ongoing HIV stigma reduction messages/activities on compassion, support and advocacy for PLHIV with their church communities [24, 27, 35]. Participants were also quite knowledgeable about HIV. However, among these and other factors hypothesized to be related to HIV stigma, only greater HIV knowledge and income were predictive of a lower HIV stigma score at baseline. Other church-population studies have also found a relationship between HIV knowledge and stigma [35, 36], and they have emphasized the role AA churches can serve in correcting HIV myths and sharing facts about HIV transmission/prevention to address HIV stigma.

No significant differences were found between intervention and comparison groups for the individual HIV items or composite scores assessed at baseline, 6-month and 12-month assessments. Also, subgroup analysis found no significant effects on HIV stigma score outcomes based on hypothesized factors. Several aspects of the study may have contributed to the null effects. Significant reductions in composite HIV stigma scores may have been difficult to achieve due to the low levels of HIV stigma measured at baseline; most of the stigma items ranged from 1.59 to 2.04 (possible range 1 to 4). However, higher levels were found for two stigma items: concern about discrimination if tested HIV-positive and PLHIV being responsible for illness. Yet, small reductions in HIV stigma scores, which trended towards significance within experimental groups at six months, and HIV stigma items occurred over time, possibly suggesting that the intervention could bring about small shifts in HIV stigma.

The process evaluation revealed that participants were highly satisfied with the TIPS intervention. Also, some of the TIPS materials/activities, particularly those (e.g., sermons, printed and video testimonials, brochures/church bulletins) delivered in church services and group ministries, were significantly related to lower HIV stigma beliefs. However, with the near-floor-level HIV stigma beliefs at baseline, an intervention with increased strength and dosage of these components and inclusion of more HIV stigma reduction strategies may be needed to shift stigma beliefs. For example, studies have shown that altruistic intervention strategies [43, 44] may contribute to reductions in HIV stigma; yet, most of these studies were individually or group delivered and have not been examined with communities of ethnic minority participants. There is more to learn about mobilizing church communities to address HIV stigma, particularly in using a CBPR-guided approach, through various church ministries to increase church reach of HIV stigma reduction strategies. These church ministry strategies could include the use of: (a) community outreach ministries (support groups, food/clothing pantry services, prayer circles) for those affected by and living with HIV; (b) church services with pastors/church leaders role modelling and promoting HIV compassion through brief plays and liturgical readings; (c) ministry group discussions on HIV stigma; and (d) self-assessments on personal HIV stigma beliefs and strategies to address one's stigmatizing beliefs. Also, future research is needed on how church/community members can be trained to serve as health advocates assisting PLHIV with linkage to and maintenance of health and social services.

This study had limitations, particularly related to methodological issues. Given that it was a pilot study, the null effects could possibly be attributed to insufficient power. Although each of the four participating churches on average had 136 participants at baseline, more churches are probably needed to detect a significant difference in HIV stigma. Further HIV stigma research with church-based interventions is needed using an appropriately powered research designs. This study also incurred significant attrition at 6 and 12 months, especially among participants who tended to be younger, male and less educated/lower income. Most of these characteristics (male, less educated/income) were highly representative of our community participants, who at times were difficult to contact due their transience and irregular contact with participating churches. Yet even with participant attrition, differential stigma beliefs between experimental groups were not detected. Also, the reliability of the HIV stigma composite measure was moderate at best in magnitude even after dropping one of the HIV stigma items from the HIV stigma composite variable. Possibly increasing the number of questions and dimensionality of the HIV stigma composite scale may enhance the reliability of this variable. Additionally, to increase understanding of HIV stigma in church settings, inclusion of religion-attributed HIV stigma measurements [35] and relevant behavioural outcomes (e.g., supportive acts similarly extended to persons with other chronic diseases) may be important to consider. For example, measures inclusive of hypothetical situations in which HIV stigma (and related compassionate acts) could occur may be more appropriate for measuring stigma in church populations [45]. Furthermore, it is possible that participants socially responded to stigma questions, especially considering surveys were completed at participating churches. Yet, baseline HIV stigma findings (and non-existent ICCs) suggest that if social responding occurred, it was similar between the randomized groups. Still, use of measurements to detect social responding among church populations may be necessary [46]. Finally, since this community-engaged study was tailored for a specific AA population and included pastors willing to participate in addressing HIV, findings may not generalize to other faith-based settings.

## Conclusions

With their reach and influence, AA churches can play an important role in changing HIV stigma beliefs in their church communities, particularly by promoting compassion and providing support for PLHIV, while advocating for elimination of injustices and discrimination against PLHIV. Rigorous AA church-based studies are needed that: (a) focus on measurement and retention issues in evaluating HIV stigma beliefs in church populations and (b) test AA church-based interventions that equip faith leaders with religiously tailored stigma reduction tools and strategies.
